# An IoT Sensing Platform and Serious Game for Remote Martial Arts Training

**DOI:** 10.3390/s23177565

**Published:** 2023-08-31

**Authors:** Karlos Ishac, Katia Bourahmoune, Marc Carmichael

**Affiliations:** Faculty of Engineering and Information Technology, University of Technology Sydney, Broadway, P.O. Box 123, Sydney, NSW 2007, Australia

**Keywords:** martial arts, biomechanics, sensing, IoT, gamification, Kung Fu, Serious Games

## Abstract

We propose a system for self-supported martial arts training using an IoT sensing platform and Serious Game that can also be extended for general sports training. In martial arts, it is important that the practitioner is correctly performing each technique to accurately learn and prevent injury. A common stance in all martial arts, but especially in Shaolin Kung Fu, is the horse stance or Mabu. With the pandemic, many more people adopted remote training without the presence of a professional trainer to give advice. Our developed LifeMat system, which is a novel IoT pressure-sensitive training mat, uses pressure maps and pattern recognition to accurately classify key martial arts postures, provide feedback on form, and detect when the user performs the technique incorrectly. This is presented in the form of a Serious Game we have developed named Kung Future that focuses on the Mabu stance as a case study. We tested 14 participants with three different feedback conditions and demonstrated that, on average, participants had higher performance, duration, engagement, and motivation when game feedback was active. Furthermore, user responses from the surveys suggested positive feedback for real-world and long-term use and scalability.

## 1. Introduction

A popular Chinese saying goes “All martial arts under heaven originated from Shaolin”, a testament to the importance of Shaolin in shaping both historical and modern martial arts [1]. Many people today know Shaolin Kung Fu through the countless movies and TV series, which in turn, have proven to create a curiosity about martial arts [2]. There are different perspectives about the origin of Shaolin. The most-widely accepted view is that Shaolin Kung Fu was taught to the monks of the Shaolin Temple in Henan, China, by the traveling Indian monk named Bodhidharma [3,4]. This early stage of Kung Fu was believed to have been mostly internal and primarily introduced for exercise. Some believe that is was Sengchou, an early disciple of Bodhidharma, that helped develop the physical component of the art along with the regular invasions of the Shaolin Temple, which caused the monks to develop methods to protect themselves [5]. The monks adapted the moves taught by Bodhidharma into Kung Fu techniques, which developed and branched out into different styles and lineages over time, which are widely practiced across the globe today [6].

### 1.1. Shaolin Kung Fu

With remote working becoming more commonplace in recent years, many people have looked to technology to assist with maintaining their fitness and learning new skills from their home. Martial arts is no exception, with people turning to video platforms and books to get started [7]. However, martial arts requires a high level of precision and attention to detail, and without the presence of a teacher or any feedback, one may be training an incorrect technique or developing bad habits or poor training, which may lead to injury. An active form of feedback is essential to understand the progression of a martial arts technique during training, especially for beginners. Additionally, gamification has proven to be a significant method for engaging users and helping people to learn new skills [8]. In particular, the field of “Serious Games” looks at the development of games for improving real-world human health and experiences. For this reason, we developed a Serious Game system that could be used to train basic martial arts poses tele-remotely through the use of physical human sensing and active feedback.

In our research, we chose to focus on an entry-level, yet essential core skill in learning Shaolin Kung Fu, the Mabu stance [9,10]. In Shaolin Kung Fu, Mabu or the horse stance is a fundamental stance for both beginners and experts. It involves standing with the feet shoulder-width apart and the knees bent, while keeping the back straight and the arms relaxed, usually by one’s side or joined in the middle. It is also the basis of many core Kung Fu techniques, highlighting its importance for practice. Horse stance is also useful for developing leg strength, stability, and stamina. It is also believed that Mabu helps to improve balance and increase overall energy flow throughout the body. Since Mabu is an isometric exercise, one typically focuses on holding the stance for as long as possible with good posture. In [9], the author explains that Mabu is based on similar principles of Wuji, a Chinese philosophy, relating to the concept of infinity or “the primordial universe”, and that the principle of “Chen”, which can be translated to “to sink”, is applied to create stability through the legs and feet while keeping the rest of the body relaxed.

Traditional martial arts training has been scientifically proven to improve balance in as little as 4 weeks [11]. Although the effects of horse stance have not been widely researched in previous articles, its concepts are widely understood by the martial arts community. Dr. Yang Jwing-Ming performed research on the White Crane style of Shaolin and explains the importance of the Horse stance in his book “*Shaolin Kung Fu and its historical background*” [12]. He ascertains the link with Wuji and breaks down the horse stance in terms of Yin and Yang philosophy. The research in [13] explains the links between Shaolin and mindfulness, highlighting the benefits of controlled breathing and body strengthening in order to maintain one’s health and prevent the onset of physical illness. An additional study in [14] demonstrated that a group training program that included Mabu training can quickly and effectively improve performance in tasks requiring lower-body muscle strength (long jump) and abdominal muscle endurance (sit-ups) in 6- to 8-year-old boys.

### 1.2. Martial Arts and Gamification

Gamification has become a popular tool for promoting learning and skill development in various domains, including martial arts. Several studies have investigated the effects of gamification on learning martial arts training, highlighting the potential benefits of this approach. The work in [15] conducted a study to investigate the effects of gamification on improving reasoning ability among elementary school students and found that gamification was a substantial contributor in improving the students’ reasoning ability, motivation, and engagement in learning. A VR-based system for learning English presented in [16] demonstrated that students had improved long-term learning and higher levels of motivation when interacting with a gamified system. The research in [17] presented a study on the use of electronic body protectors and gamification in the sport of Taekwondo. The results showed that training with an electronic body protector improved the participants’ condition and technical skills and also helped them learn the correct use of the techniques. We have also previously conducted research into Taekwondo, where we used an system to analyze the unique kinematic properties of the Sine-wave punch with the future goal of analyzing the correct technique and gamifying one’s skill level [18]. A systematic review of gamification in sports highlighted the benefits of gamification by allowing a user to train despite the weather conditions whilst improving his/her motivation and performance [19].

Overall, the literature suggests that gamification can enhance the learning experience and promote skill development in martial arts training. Gamification elements, such as feedback, rewards, challenges, and social interaction, can promote intrinsic motivation and a sense of accomplishment, which can lead to increased engagement and skill development. Future research could explore the design principles for effective gamification in martial arts training and investigate the long-term effects of gamification on skill development and retention.

Martial arts training presented through immersive media has been explored in various popular video games such as the Eyetoy Kinetic Combat on the Sony Playstation 2 [20] and the Fighter Within on the Xbox One [21]. These games use a camera and depth sensor to track a user’s motion in the real-world in order to control a virtual character in-game. Similarly, the research in [22] used a Kinect device and a threshold posture-detection method as a whole-body controller for an AI-based fighting game called FightingICE. Kinect-based controllers have been widely explored, but often lack the accuracy needed to classify and analyze finer techniques in the martial arts. Perhaps the most-well-known interactive gaming device for health and training is the Wii Balance Board by Nintendo. This widely popular system was introduced as a way to measure your weight and fitness levels at home through the use of multiple scale-based mini-games. It garnered a wider audience than the typical video game player and has found use outside of the gaming industry in other areas such as balance assessment [23,24,25] and rehabilitation [26,27,28]. In rehabilitation, it has been used to assist patients with stroke [26], multiple sclerosis [27], and vestibular impairments [28]. The Wii Balance Board is, however, limited by its small size, elevated platform, rigid structure, and hard surface. This makes it unusable for most martial arts (and sports training) as the forms in martial arts require a stance much wider than that provided by the Wii Balance Board platform and are all practiced while grounded on the floor. For this reason, we have developed our system specifically to account for a large surface area for movement while keeping elevation minimal to ensure the user is grounded and can perform his/her technique naturally.

The research in [29] developed a game called Kick Ass Kung Fu, which uses computer vision techniques to control a virtual avatar to defeat in-game enemies in a 2D scene. They opted for a camera-based approach because, at the time, floor sensors were quite expensive in comparison, and computer vision allowed for full-body tracking. With our eTextile approach, we have managed to develop a low-cost, affordable IoT sensing mat for martial arts training. Furthermore, while computer-vision-based approaches are ideal for full-body tracking, it is also important to evaluate the precise pressure distribution and balance information of the feet, which is essential in proper training of martial arts stances and postures. The study in [30] attempted to resolve camera field of view limitations by using two Kinect sensors to capture the 360° motion of the user while performing the Tantui form in Kung Fu. The research also demonstrated that using augmented reality to learn martial arts enhanced student motivation. Reference [31] proposed an interesting method to solve the 3D perspective problem in virtual martial arts training. They took advantage of augmented reality capabilities to project a series of virtual coaches around the user, which shows the relative posture for a technique at a given angle. Since Tai-Chi Chuan involves much turning, the 3D perspective is important to avoid issues related to camera occlusion. Any experienced martial arts practitioner will also emphasize the importance of the correct weight distribution for different stances and techniques. The research in [32] used a Mixed Reality headset and in-sole pressure sensors to teach the correct weight distribution when performing basic Tai-Chi Chuan techniques. However, the calibration of the weight distribution is heavily dependent on user performance, and the variability of the calibration methodology exposes it to significant human error. Furthermore, the system cannot detect the relative foot position and, hence, cannot determine the posture and COP properties. The system we propose in this research uses the advantages of our pressure sensor matrix approach for real-time mapping of both feet relative to each other, COP parameters, and user posture, to solve some of the limitations evident in previous works.

### 1.3. Physical Human Sensing

Our previous research investigated various IoT interfaces and novel sensor technologies for improving human posture in the workplace [33,34,35,36]. We extended this research with gamification elements and feedback in the form of a social robot [37]. All these studies demonstrated that active feedback components were beneficial for training healthier habits and effectively improving workplace performance. We also analyzed human motion in soccer, where we used a smart ball and vision-based system to score and classify the knuckleball effect [38]. It highlighted the phenomenal effects slight changes in human biomechanics can produce and the insight that can be gathered from joint sensor capabilities. In a more-dynamic scenario, we also used vision-based sensors and vibration sensors to evaluate different human skills in a trampolining environment. Based on our previous research, we determined that a combination of a pressure-sensitive training mat and vision-based system would be an optimal testing setup for an isometric technique such as the Shaolin Mabu stance. Our developed posture models were influenced by our previous works with human sitting posture, human motion analysis, and sports skills experiments. The active feedback methodologies were derived from positive results identified in our previous intervention studies [34,35,37] and used as a basis for developing the app interface and gamification elements in this research.

**Figure 1:**
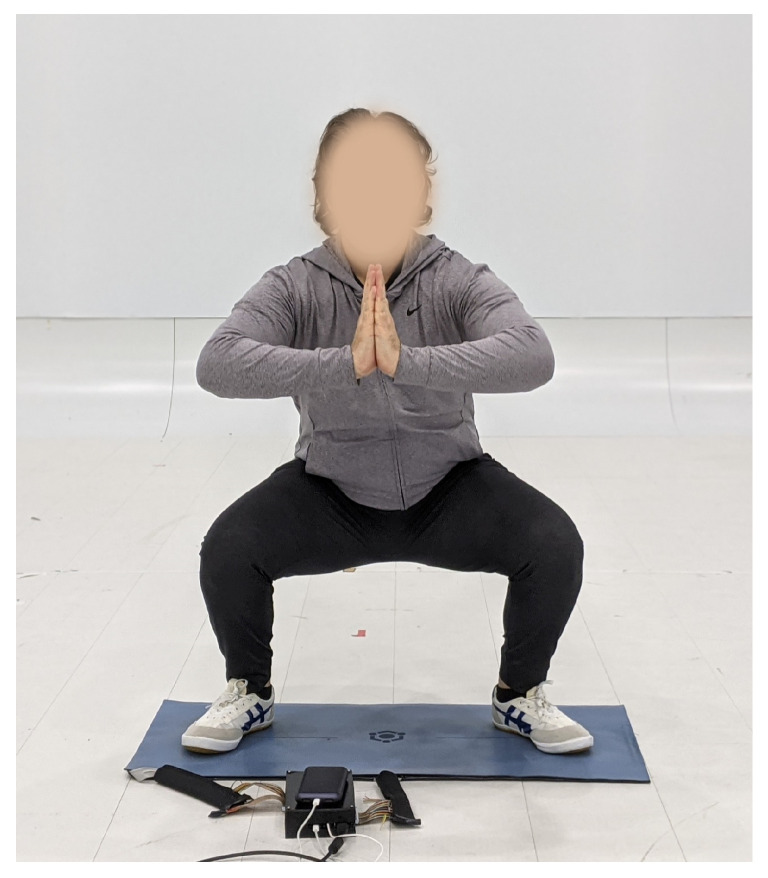
Example of Mabu stance.

This paper presents research into the effectiveness of an IoT sensing platform and Serious Game for self-guided remote martial arts training. We focus on the entry-level, yet fundamental technique of Mabu in Shaolin Kung Fu which is depicted in Figure 1. In this research, we consulted with experts in the art of Shaolin Kung Fu to design the app interface and to ensure our experimental methodologies are valid.

Our hypothesis was that, through the use of an IoT active feedback system and Serious Game, one can improve his/her basic martial arts techniques remotely, without the need for a trainer to be present. It is critical to note that our proposed system is designed for introductory-level martial arts techniques to assist entry-level practitioners to accelerate their training in the early phases. Martial arts is a complex collection of physical skills that require the need for professionals to advance in later stages, and the value of professional human feedback is intangible.

### 1.4. Contributions

Our contributions to the research field of sensors, the IoT, and health monitoring are:

- The development and evaluation of a novel pressure-sensitive mat that is embedded into a standard training mat for accessible and affordable human activity analysis.
- The implementation of a novel type of sensor that has been specially manufactured to meet the design requirements based on performance testing from previous research in IoT-based health monitoring.
- The development and evaluation of the first-ever platform for remote martial arts training that uses IoT sensing.
- The first IoT-based investigation of the Mabu posture in Shaolin Kung Fu.
- A scalable sensing platform that combines gamification and feedback elements for guiding human performance during training.
- A preliminary user feedback survey and directional results on system effectiveness, which will guide further investigations in our future work and the research community.

### 1.5. Research Outline

This paper is structured to firstly introduce the background of Shaolin Kung Fu and its origins. We then provide a literature review on previous works in the fields of martial arts, gamification, IoT human sensing, and Serious Games. The paper then outlines our methodology for the system design and development, with insight into the user-centered design and relation to our previous work in the field. The experimental outline and results are then presented. Finally, we evaluate the results in our discussion in line with our research objectives and hypothesis while also considering system limitations and future work.

## 2. Methods

**Figure 2:**
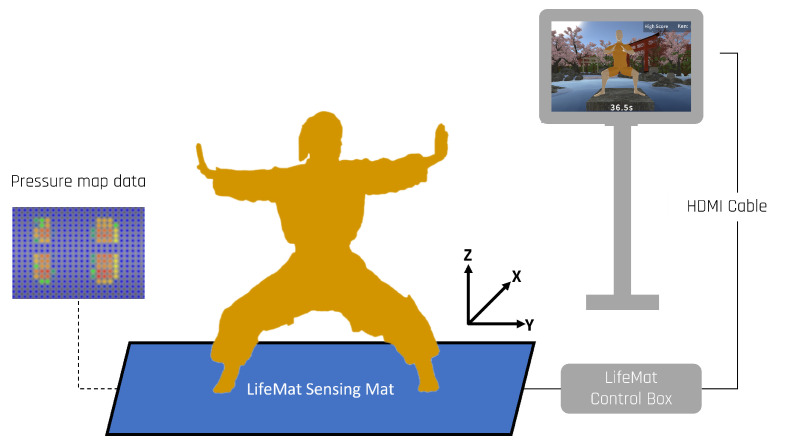
Kung Future system overview.

Our system is comprised of a Serious Game called Kung Future, a large IoT pressure-sensitive training mat called LifeMat, a LattePanda single-board computer (SBC), and a high-definition (HD) monitor display. The system setup is depicted in Figure 2.

### 2.1. Design Requirements

When considering the development of our system in relation to our research question, we outlined important design requirements that we strictly followed throughout the research project. The design requirements are as follows:

- Understanding the biomechanics of the Mabu stance in Shaolin Kung Fu;
- Consulting with a professional trainer or “Sifu” during development and experiments;
- Developing an interface for measuring balance and foot position;
- Using a large sensing surface to accommodate the wide stance of Mabu;
- Applying a Serious Game to test the effect of gamification;
- Developing a system that could be deployed for remote self-supported training.

Based on these design requirements, we firstly acquired a comprehensive understanding of the foundations of Shaolin Kung Fu and Mabu through an extensive literature review and analysis of existing biomechanical models. We then implemented the LifeMat pressure-sensing surface, ensuring that it could provide pressure-sensitive data for a wide stance like Mabu. We then developed the Serious Game app called Kung Future, while consulting with a Shaolin Kung Fu Master. Finally, we evaluated our proposed system in a user study.

### 2.2. The Mabu Stance

In the development of our system, we consulted with a Sifu in Shaolin Kung Fu to verify the biomechanics of the horse stance that was presented in our literature review. Mabu can be performed by following these steps, which are a combination of those defined in previous research [9] and instructions obtained from Shaolin professionals:

1. Stand in a wide stance while maintaining good balance.
2. Point the toes slightly outwards at an angle of approximately 15–20°.
3. Bend the knees until your quadriceps are parallel to the ground; a Bo Staff should be able to rest across the upper legs without falling (a high Mabu variation can also be adopted by not squatting as low).
4. Rest your fists by your side at waist height while keeping your arms relaxed and elbows pointing back (alternatively, the palms can be joined in front of the chest in a prayer-like gesture).
5. Keep your back straight and equally balanced between left and right.
6. Breath in slowly through the nose and out through the mouth.
7. Maintain the Mabu position while keeping a good balance and straight back and not moving the feet as if they were the roots of a tree.

These are the same steps presented to participants in the tutorial video during the feedback experiments of this research.

### 2.3. LifeMat System

The LifeMat is an Internet of Things (IoT) pressure-sensitive interface embedded into a standard training mat interface and is produced by Lifeform AI [39]. The device is flexible, thin, and lightweight, allowing a practitioner to perform his/her techniques in a natural setting and grounded surface. Its dimensions are 1000 mm long, 500 mm wide, and 3 mm thick. This large sensing area allows for accurate analysis of human performance in various sports, physical arts, and exercises, which are not possible using conventional balance boards or force plate systems. In our configuration, the LifeMat can sense 924 individual pressure points in 12 bit resolution in real-time.

The LifeMat uses piezoresistive technology and a pressure sensor matrix formation for measuring human posture. This provides us with insight into the position, rotation, and location of each individual foot on the mat. It also provides information such as foot pressure distribution, foot balance parameters, and fine data such as toe and heel placement. Using this information, we can determine the user’s posture and motion in real-time and apply this to Shaolin Kung Fu.

**Figure 3:**
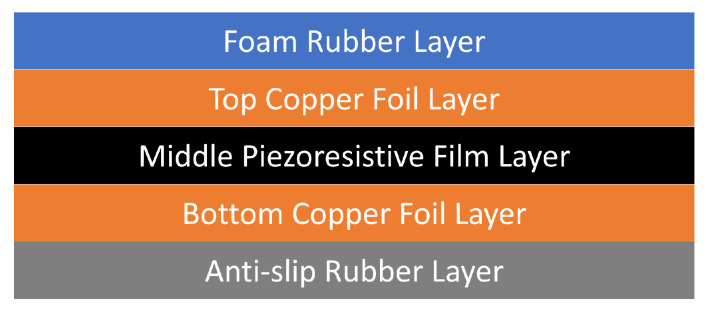
Sensing system layer description.

Figure 3 illustrates the sensing system and its multi-layer composition of conductive and resistive materials. During our pilot experiments, we tested several types of conductive flexible materials including conductive fabric (woven and knitted), conductive ink printed on flexible plastic, and conductive foils. Of these, copper foil was the most effective for this application due to its consistency, flexibility, lifespan, and minimal resistance across its length. It demonstrated close to 0 resistance across the entire 1000 mm length of the copper foil strips, which was the maximum length used in this system. This provided significantly improved sensing consistency when compared to other materials tested such as printed silver on flexible plastic, which would exhibit varying results depending on the distance of the sensing point from the PCB connection.

For the piezoresistive layer, typically systems would use Velostat/Linqstat, but it is expensive to produce, difficult to source in large quantities, and is becoming more scarce. With a real-world implementation in mind as part of our design criteria, we sought an alternative that was more cost effective to keep aligned with our aim of providing an accessible remote training platform. We installed the middle piezoresistive layer using carbon-filled polyurethane (PE) film sheets that were specially sourced based on our physical and electrical design specifications. The piezoresistive film was placed carefully to ensure the top and bottom conductive layers would not come into direct contact. The middle layer was designed to be 0.1 mm in thickness and flexible in form. The conductive and piezoresistive layers combine to produce a flexible pressure-sensing matrix, which allows our sensor to be customized based on the sensing resolution, size, and shape required.

The LifeMat sensor mat is connected to a custom-made PCB, which processes all the data from the pressure sensor matrix and communicates this information to the Kung Future Game. The PCB contains a series of multiplexers and shift registers, which switch between the rows and columns of the conductive sensors to be able to rapidly scan the entire sensor matrix for analog pressure values. The PCB utilizes a voltage divider principle, which is used in conjunction with the piezoresistive effect to measure a range of pressure values. The PCB includes modular inputs for 50-pin ribbon connectors so that any combination of up to 50 rows or 50 columns can be input to adjust the number of sensors in the LifeMat system. The PCB is connected to the Latte Panda SBC and communicates via USB-C protocol to a laptop or computer. The LifeMat can also operate wirelessly using Bluetooth Low Energy or WiFi communication for data transfer, but for the purposes of this research, we operated it in a wired connection state. The Latte Panda SBC is connected to an external HD monitor display via an HDMI cable. The LifeMat device can be powered through a standard 5V power supply.

We developed the LifeMat with IoT capabilities to allow it to store and track individual user performance over time by communicating with an online database hosted on the cloud. This is optimized when communicating through the Kung Future App, which connects to the databases to retrieve user statistics and improvements over time.

The LifeMat primarily uses its inbuilt WiFi for data communication when operating in wireless mode. In this research, the system operates through a wired connection and directly accesses information through the WiFi module of the LattePanda SBC.

### 2.4. Kung Future Game

**Figure 4:**
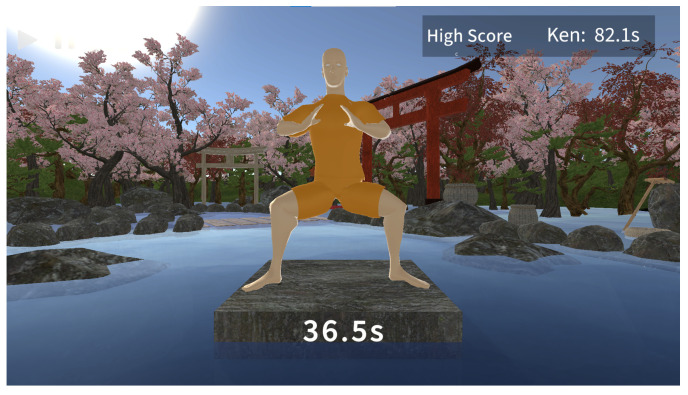
Kung Future main game interface.

We developed the Kung Future Game using the Unity 3D platform [40]. In the development of the game, we used real-world video data to create the 3D model of the virtual coach for the beginners to follow. The game scene was designed to induce a relaxed focus state, which is the goal for true Mabu training. To achieve this, we combined elements of water, trees, and flora to create a Zen garden setting. Additionally, we implemented an instrumental calming background music. The game interface is illustrated in Figure 4.

The game operates by firstly verifying the initial Mabu posture of the user based on our developed biomechanics model for the LifeMat system. It does this by validating the foot position, rotation, and pressure distribution of the user before allowing his/her to begin Mabu training. It does this through the data received by the LifeMat system. If the user’s foot placement or posture is imbalanced or incorrectly oriented, the game will provide a warning along with feedback on how to correct his/her stance before beginning.

Once the user is in the correct Mabu posture, the training begins and the timer starts. In training mode, a timer shows how many seconds the practitioner has correctly held the Mabu posture for. If the user breaks his/her stance, the LifeMat system detects a change in posture and sends a signal to the Kung Future app, which then causes the game to automatically stop the timer and provide feedback. The stance can be broken in several ways. Either the practitioner loses his/her balance or leans too much in a particular direction, gives up his/her stance due to fatigue, moves his/her feet to an incorrect position, has a major shift in his/her center of pressure (CoP) due to imbalance, or rises up to a higher stance, which may be considered a high Mabu stance.

### 2.5. Gamification and Feedback System

We considered several modes of feedback when designing the system. Previously, we used vibrotactile feedback [34,35], emotive and kinetic feedback [37], and visual feedback [38] to augment the human performance in different scenarios. For this system, we decided to test visual feedback in a game-type interface due to its potential for long-term user engagement and ability to communicate information to the user in an intuitive and comprehensive manner. We also considered the aim of the system to be used for independent and remote training. For this reason, a game-type interface with a basic reward system would be ideal for communicating instant beginner-level feedback, while displaying progress over time and keeping the user engaged for a long-term training program.

During training mode, the user receives in-game feedback on his/her Mabu posture through the virtual Master’s mirroring, which reflects the user’s balance and posture. The visual feedback is instant and allows a user to correct his/her posture without training bad habits or the incorrect technique. This is especially important in remote training, where a human coach is rarely present.

**Figure 5:**
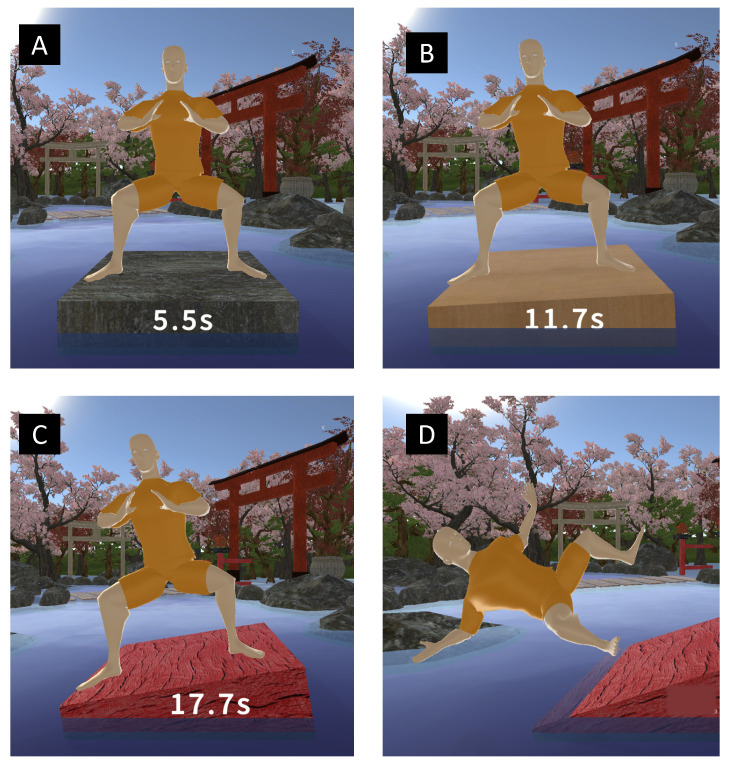
Kung Future game feedback system. (**A**) balanced Mabu condition, (**B**) slightly leaning Mabu stance, (**C**) significantly leaning Mabu stance, (**D**) extremely imbalanced stance breaking Mabu.

Within the Kung Future game, we used feedback in the form of a tilting platform that would lean to either side depending on the real-world posture of the user. If the user would lean too much to one side, he/she would break his/her Mabu stance and the virtual character would fall into the water. Simultaneously, the platform would change from a grey color in the balanced position to a yellow color when the user was slightly leaning and a red color when the user was very imbalanced. In this feedback mode, the goal was to keep the virtual Sifu (avatar) on the platform above the water for as long as possible by maintaining the correct and a balanced Mabu stance. An example of the in-game feedback mechanic is illustrated in Figure 5.

The instantaneous pressure matrix snapshot of the LifeMat can be denoted as $M_{c}$ for the calibration frame and $M_{t}$ for the real-time frame during training. Using the pressure matrix *M*, we calculate the COP of the left and right feet, which are $COP_{l}$ and $COP_{r}$, respectively. We also compute the overall $COP_{o}$ using the same method. During gameplay, we analyze the real-time matrix $M_{t}$ for postural imbalances by checking the pressure distribution values in the individual feet and between the left and right foot. If the pressure is imbalanced, then a virtual flag is triggered and the user is deemed to have released the Mabu posture, in which case, the timer stops. We also use the matrix $M_{t}$ to check the foot position and orientation on the LifeMat to ensure the user remains in the correct posture during training. If the user shifts his/her foot to an incorrect orientation or position, then a virtual flag is triggered and the timer stops.

During training, we also use the COP value to check if the user has released his/her Mabu posture by shifting too much from his/her calibrated origin point. We determine this by checking the real-time values of $COP_{l_{t}}$, $COP_{r_{t}}$ and $COP_{o_{t}}$ with their calibrated reference values. The COP values are 2D coordinate values on the LifeMat coordinate frame and are projected as *x* and *y* components, which can also be represented during training mode as $COPx_{o_{t}}$, $COPy_{o_{t}}$, $COPx_{l_{t}}$, $COPy_{l_{t}}$, $COPx_{r_{t}}$, and $COPy_{r_{t}}$. We define a range for valid Mabu postures for that user as all values that are within the circular area defined by the COP values at the calibrated origin point and a radius of α. The value of α is a float value and was determined during our pilot experiments through monitoring the *COP* changes of a user when he/she was upright and leaning to each side. The defined circles are set for the left foot, right foot, and overall *COP* of the user. If the COP value of the left foot, right foot, or overall *COP* is outside of the valid circular region, then the user is deemed to have shifted too far from a good Mabu posture, causing a virtual flag to be triggered and the timer to stop. The virtual flags are triggered if any of the *COP* values exit the good Mabu region. The state of good Mabu is defined as the matrix $M_{s}$ for a given user, and the state of poor Mabu is defined as the matrix $M_{p}$ for a given user. This is highlighted in Equations (1) and (2) for the case of $COPx_{o}$ and $COPy_{o}$, but the same equations can be applied to $COP_{l}$ and $COP_{r}$.

(1) $$M_{s}\left\{\begin{array}{cc} x: & COPx_{o_{c}}-\alpha <COPx_{o_{t}}<COPx_{o_{c}}+\alpha ;\\ y: & COPy_{o_{c}}-\alpha <COPy_{o_{t}}<COPy_{o_{c}}+\alpha . \end{array}\right.$$

(2) $$M_{p}\left\{\begin{array}{cc} x: & COPx_{o_{t}}<COPx_{o_{c}}-\alpha \;\overset{}{\underset{}{⋁}}\;COPx_{o_{c}}+\alpha <COPx_{o_{t}};\\ y: & COPy_{o_{t}}<COPy_{o_{c}}-\alpha \;\overset{}{\underset{}{⋁}}\;COPy_{o_{c}}+\alpha <COPy_{o_{t}}. \end{array}\right.$$

During calibration, the calibration frame $M_{c}$ is analyzed, and if all the criteria for good Mabu posture are not met, then the user is instructed to redo the calibration until the posture is valid. The sensor matrices $M_{c}$ and $M_{t}$ are both processed to be used as inputs to the modeling and feedback algorithms. At first, the data are filtered using a high-pass filter that attenuates pressure values below a float value of β. The value of β is determined from experimental testing and is necessary to account for the weight of the top foam layers when no user is present. The filter is used to set the true zero matrix of the pressure mat and to also minimize any noise that may be present. After the matrices are filtered, the data are segmented by separating the sensor points into the left foot matrix $M_{l}$ and the right foot matrix $M_{r}$, which are the left and right half of the LifeMat, respectively. This is specific to this application of the LifeMat since the user is expected to perform an isometric posture that requires his/her left foot to be on the left half of the sensor mat and his/her right foot to be on the right half. The matrix $M_{t}$ is then used to compute the overall *COP* and positioning parameters. $M_{l}$ is used to compute the left foot *COP* and positioning parameters, and $M_{r}$ is used to compute the right foot *COP* and positioning parameters.

The reward system we developed for Kung Future was a simple level-up mechanics commonly used in many popular video games. The practitioner would begin at Level 0 and then ascend to the next level for every 10 s he/she held the Mabu posture correctly. Furthermore, we grouped the levels based on recommended durations to hold Mabu based on previous literature. The beginner group was Levels 0 to 6, the intermediate group Levels 7 to 12, the veteran group Levels 13–18, and the expert group Levels 19 and onwards. All participants had their total durations, final level, group association, and overall ranking, displayed and recorded on the leaderboard.

The primary goal of the game is to introduce the Mabu posture and help a beginner practice the technique correctly on his/her own and in a remote setting. This is intended to be used in addition to regular training classes. Experts may also use the system to evaluate the fine points of his/her technique such as stability, balance, and foot placement, which may be difficult to self-review during solo training. Additionally, since the focus of this research was to evaluate gamification for remote training, we included a leaderboard feature for the longest time spent in the correct Mabu stance and included all users on the leaderboard in order of their ranking. Finally, we added another level of competition to the system by providing monetary prizes (gift cards) to the top scorer of the experiment. The effectiveness of gamification elements for training was analyzed in our post-study survey.

## 3. Results

**Figure 6:**
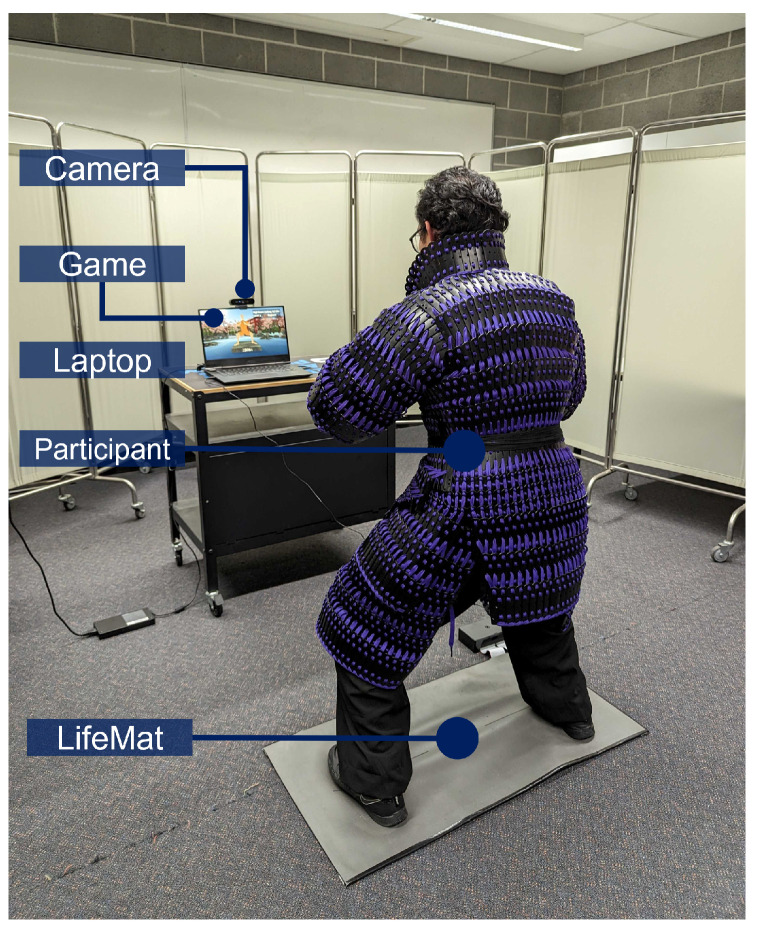
User intervention study experimental setup.

The main focus of our research was to evaluate the effectiveness of our IoT platform and Serious Game for remote training in martial arts. In order to test this, we first validated the feasibility of the LifeMat system in collecting data for isometric martial arts stances. We then evaluated the effectiveness of our system through an intervention study which is depicted in Figure 6. Finally, we surveyed each participant on his/her experience and learning through using our developed system.

### 3.1. Participants

We conducted experiments on 14 healthy adult participants with previous martial arts experience and that were part of an active martial arts club and weekly training program. Prior martial arts experience was a requirement for the experiment as the subject needed to have a basis of training history to be able to compare the different modes of feedback with reference to traditional training. No prior video game experience was required. There were 11 male participants and 3 female participants. The mean height was 172.83 cm with a standard deviation of 8.19 cm. The mean weight was 83.0 kg with a standard deviation of 23.79 cm. Ethical approval was obtained for this study, and consent was obtained from all participants prior to beginning the experiment.

### 3.2. LifeMat for Martial Arts Feasibility Experiment

We tested the LifeMat’s capability for being used as a device for martial arts training by measuring its accuracy and precision in detecting static parameters. These included the foot position, distance between the feet, and left and right balance. These parameters are all used within the Kung Future app; hence, it was critical to confirm the accuracy of the readings. We used our developed app to generate heat maps and CoP readings to cross-validate each user’s starting posture with the Mabu biomechanical model outlined by previous literature.

In particular, our analysis included the following criteria for evaluating correct Mabu stance by the LifeMat system:

- Left and right pressure distribution should be equal and balanced.
- Feet should be along the horizontal center-line of the LifeMat.
- Feet should be wider than shoulder-width apart.
- Each foot should be slightly pointing outwards between 0 and 45°.
- Sole and heel pressure should be balanced.

**Figure 7:**
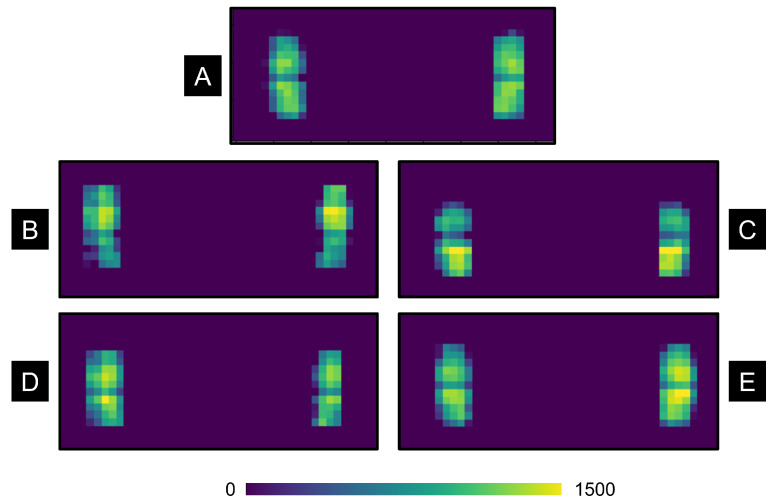
Example of filtered heat map results as visualized by the LifeMat for (**A**) correct Mabu Stance, (**B**) incorrect Mabu stance—leaning forward, (**C**) incorrect Mabu stance—leaning back, (**D**) incorrect Mabu stance—leaning left, and (**E**) incorrect Mabu stance—leaning right.

We tested these criteria by having a professional perform Mabu correctly and incorrectly on the LifeMat system for 10 trials in the correct posture and 10 trials in each of the incorrect postures by leaning in various directions. The LifeMat recorded the heat maps and CoP data in real-time. Examples of the heat maps for both correct and incorrect Mabu variations are depicted in Figure 7.

### 3.3. Gamification of Training Experiment

After validating the LifeMat system’s capabilities of detecting stance parameters, we proceeded to conduct the user experiments for evaluating the effectiveness of gamification. Each participant was asked to perform the Mabu stance for as long as possible in 1 of 3 conditions, and the target of this experiment was to compare the duration of the performed Mabu stance in the different conditions. The order of these conditions was randomized to avoid fatigue-related bias. The conditions tested were:

(i) NFB: no feedback.
(ii) CFB: watching a clock with a count up stopwatch.
(iii) GFB: feedback from the Kung Future game.

Each participant was given a 5 min break between conditions to prevent fatigue. During the experiment, privacy dividers were used to replicate the feeling of remote training to ensure a realistic use case scenario was recreated and that the test results would be accurate. During each condition for each user, we recorded a front-angle and side-angle video of the user, pressure sensor matrix data, detected posture, foot orientation information, and total time in correct Mabu posture. A post-experiment survey was given to each participant after the experiment concluded.

Prior to the experiment, each participant watched a 3 min video tutorial on how to perform the Mabu stance. During the experiments, the app would firstly verify that the participant was in the correct Mabu stance before starting the game. Once the game had started, the app would automatically detect when the participant became unstable and broke out of the Mabu stance. At this time, the timer would stop and all data collection would cease. A sound notification would also play to notify the user that he/she had exited the Mabu stance and that the trial was over. The participant would then be given a 5 min break before proceeding to the next condition until all 3 conditions had been fulfilled.

In Conditions (i) and (ii), the participant does not see the Kung Future interface, but the system still alerts him/her with a sound notification once he/she has broken the Mabu stance. In Condition (iii), the early warning signs of an improper technique are shown through the feedback on-screen. This informs participants on how to correct their technique before they break the stance and the timer stops.

**Figure 8:**
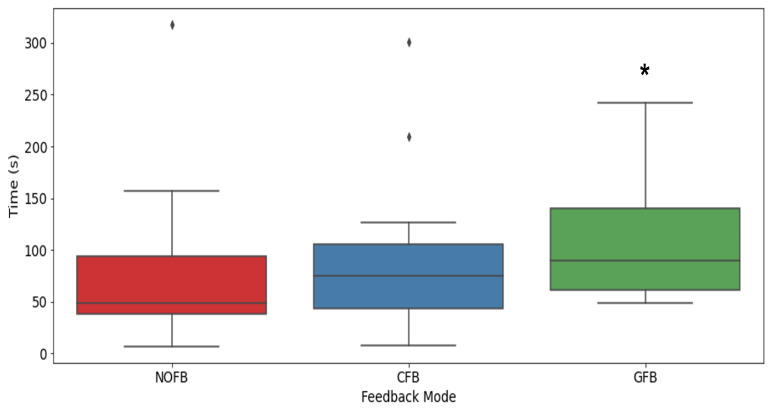
Gamification feedback experiment results of the total time in correct Mabu form for the three feedback conditions of (i) NOFB: no feedback (ii) CFB: clock feedback, and (iii) GFB: game feedback. * p<0.05 (Mann–Whitney U-test). Diamonds represent outlier scores for each condition.

The summary of the total duration for each condition across 14 participants is shown in the box plot in Figure 8. We include a Supplementary Video (Video S1) which provides an example of the gamification of training user study conducted in this research.

### 3.4. Serious Game Feedback Survey

There is no standard method of evaluating a Serious Game, but a framework proposed by [41] based on Activity Theory and extended by [42] has shown promise in surveying user experience. We adopted a similar framework for this research and modified the statements to be suitable for the Kung Future app while including questions related to our own research questions and hypothesis. A five-point Likert scale was employed to assess end-users’ level of agreement with each statement, where 1 was equivalent to “Strongly Disagree” and 5 was equivalent to “Strongly Agree”. We included questions to assess gaming experience and interactions in a virtual environment based on the survey items proposed by [43]. We slightly modified the questions to be relevant to the Kung Future Game. The list of questions used and the response results are tabulated in Table 1.

The survey consisted of three sections, which focused on (1) participant background (2) Serious Game experience survey, which used a scale rating system, and (3) a user feedback survey on gameplay and interaction, which consisted of open-ended questions. The participant background survey is tabulated in Table 2 with a summary of the results for each question. The details of each question are discussed in the next section. The Serious Game experience survey is tabulated in Table 1 with an overview of results for each individual question. The user feedback survey on gameplay and interaction is tabulated in Table 3 with a list of the open-ended survey questions. A summary of the feedback is presented in Section 4 of this paper.

## 4. Discussion

The results from our feasibility study are shown in Figure 7, which shows representative heat map data of the LifeMat pressure sensor matrix for the correct Mabu posture, leaning left in Mabu, leaning right in Mabu, leaning forward in Mabu, and leaning back in Mabu. The heat maps demonstrate that the LifeMat is capable of detecting imbalances and foot position. This is especially evident in the heat map changes when leaning in various directions.

Therefore, the LifeMat displayed the capability of detecting even very fine sways in upper body posture. This was also evident in the successive feedback study, which demonstrated the sensitivity of LifeMat. During the feedback experiments, one participant was detected to have exited the Mabu posture due to slightly adjusting his/her shoulders. According to the participant, he/she was not swaying, but the LifeMat had detected a leaning motion and a change in left foot CoP. Upon cross-validating with the video, the participant realized that he/she indeed had adjusted his/her shoulder position by slightly leaning forward in the process. The LifeMat system thus demonstrated high sensitivity in detecting very fine changes in body posture. This is due to our highly refined posture-modeling algorithms, piezoresistive sensing interface, and sensor resolution. From a system feasibility perspective, this highlights the effectiveness of the LifeMat’s pressure-sensing capabilities. For long-term use of the system as a game input or training interface, we envision including a threshold modifier for adjusting the difficulty level of the posture clusters.

The results from the intervention study are shown in Figure 8 and show a comparison of the average total duration (seconds) in Mabu for all participants in each condition. From the results, we can clearly see that the condition where the participants were receiving feedback from the Kung Future app had the highest performance in Mabu posture in terms of correct technique and time duration, and this result was significant (p<0.05, Mann–Whitney U-test). Even with randomized trials, it was clearly observable that the game feedback trial would have the highest performance result among participants. Participant feedback explained that, by concentrating on the game feedback, they had something to focus on and attempted to overcome this, which increased their motivation. Furthermore, the social and challenge features are more distinctly presented and appreciated in a visual gaming interface. Additionally, the clock feedback (CFB) ranked the second-highest in terms of participant performance in the feedback conditions. We observed that participants would mainly focus on the timer when they were struggling and would try to hold their Mabu stance by being motivated to beat a certain score on the leaderboard or place in the top three positions, with some participants displaying higher competitiveness than others. The CFB timer display provided a visual guide for users to refer to when training, and this could be attributed to the overall improved performance when compared to the NFB condition. From the game feedback and clock feedback conditions, we could also evaluate that having a visual interface increased participant performance due to the visual guide being a motivating factor. The survey results discussed later in this section also highlighted the increased motivation and engagement of participants when feedback was provided. Since participants were aware of their previous scores and the leaderboard scores during the experiments, having a timer present (CFB and GFB) gave an indication of how long they have currently held Mabu and how much longer they would need to surpass the next score. This competitive factor between individuals was clearly more present in the CFB and GFB trials and may be attributed to the increased performance results in our Serious Game.

Among the top three scorers that were able to hold the Mabu stance for the longest duration in the correct posture, all three practiced a form of Kung Fu and had some knowledge of the Mabu stance. However, none of the participants of the experiment were regularly training on the Mabu stance in the months prior to the experiments. The top scorer mentioned that he/she had practiced the stance for 3 years in the past and, after performing the experiment, would once again include the Mabu stance in his/her martial arts training routine. It was evidently observable that participants with Kung Fu experience that were more familiar with the Mabu stance performed better in the feedback experiments. Participants from the other styles of martial arts each had their own unique stances that are fundamental to their forms.

We also considered that, depending on the order of the conditions, a participant’s best-performing trial would be his/her first trial since he/she may experience fatigue in the latter feedback condition trials. However, another aspect we considered was increased performance due to familiarization through the trials. Since the trials were randomized across all participants, it was difficult to make a fundamental assumption on these two effects, but it is an important consideration to take into account in future experiments.

In evaluating the participant background survey results, several important factors were highlighted. Firstly, it is notable that all participants answered “Yes” to often playing video games in Survey Item 1.5. Even though this was not a prerequisite for the study, it was interesting to observe that all participants were both part of a regular martial arts training club and also often played video games. This may have contributed to some of the interaction responses in the latter surveys, where, depending on individual video game experience, an individual may be either embracing of gamification elements or critical of the game mechanics itself due to high standards.

**Figure 9:**
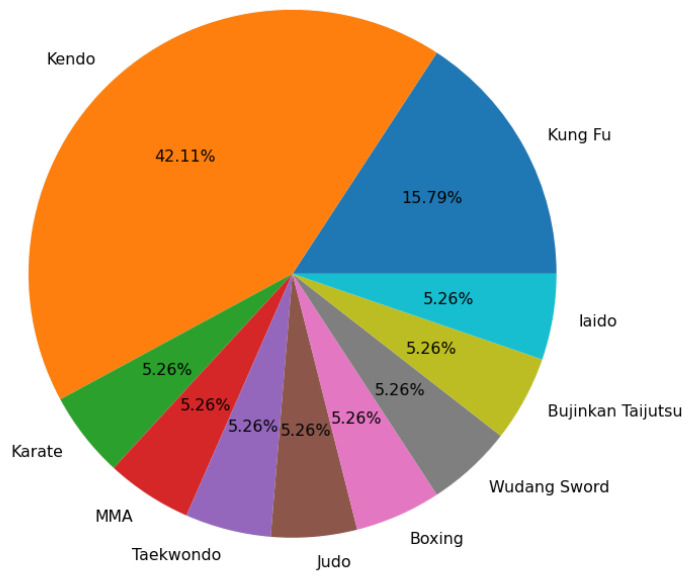
Survey of all participants’ martial arts training experience (labels may not sum to 100 due to label rounding).

The results of Survey Question 1.6 indicated that 0 participants had a low activity level, 11 participants had a moderate activity level, and 3 participants had a high activity level. The results from 1.7 also further reflected this with all 14 participants practicing a particular style of martial art. The individual martial arts practiced are displayed in Figure 9 and show a diverse range of styles. Finally, the results of Survey Question 1.8 showed that only 6 of the 14 participants practiced a sport alongside their martial arts training. The sports practiced often supplemented the martial arts training with answers that included "weightlifting” and “swimming” for strength and stamina training. When we consider the results from Survey Questions 1.6, 1.7, and 1.8 combined, it is clear that all participants had a mature understanding of martial arts training and fitness, with each participant having a unique combination of martial arts style, fitness levels, and sports training.

The results in the Serious Game experience survey showed the mean and standard deviation for each question with lower values relating to “strongly disagree” and higher values relating to “strongly agree”. In most cases, a higher value indicates a positive response towards a survey item. Survey Items 2.1, 2.2, and 2.3 were used to analyze the overall participant’s gaming efficacy. The results of Survey Item 2.1 indicated a slightly neutral score of 3.33 for gaming skill level among participants with a standard deviation of 1.23. This highlights a broad range of gaming skill level across participants; however, according to Survey Question 1.5 from the participant background survey, all participants answered “Yes” to often playing video games. This indicates that all participants are familiar with gaming interfaces, although there is a broad range of skill levels. Furthermore, the results of Survey Item 2.2 showed a high mean score of 4.50 and a low standard deviation of 0.67, with most participants stating they can figure out most video games. This is related to the learning curve of video games and individual gaming intelligence. In the design of the Kung Future app, we emphasized the aspect of making the mechanics and interface as intuitive as possible to allow for a short learning curve. Most participants were able to understand the concept of the game after following the tutorial video or by playing a single round of the game. Survey Item 2.3’s results showed a low mean score of 2.00 and a standard deviation of 0.85, highlighting that most participants were able to improve their performance while playing video games.

Survey Items 2.4 and 2.5 related to the experiential attitude of the participants. Survey Item 2.4 had a very high mean score of 4.58 with a standard deviation of 0.51. All participants selected either “Agree” or “Strongly Agree” to this question, indicating that their training experience was entertaining. In a similar manner, a very positive response was observed for Survey Item 2.5 with a mean score of 4.33 and a standard deviation of 0.65, emphasizing that, overall, participants found the experience very enjoyable. This is a positive indicator of our developed system in creating an entertaining and enjoyable experience for users, which is an important component of retaining player motivation and replay value. When developing the Kung Future app, we aimed to create an experience that was both engaging and informative with scalability for long-term use. We especially focused on using in-game mechanics such as character growth, leveling up, and leaderboards, to increase the replay value of the Kung Future game. During the experiments and across the survey results, it was evident that participants reacted positively towards the use of gamification in martial arts training. As with real-world training, consistency is key, and a user will obtain more training value through using the app to learn a new skill, train, improve through time, and track their improvements.

The set of Survey Items 2.6, 2.7, and 2.8 relates to the instrumental attitude of the user experience. Interestingly, Survey Item 2.6 had a neutral score of 3.50 with a standard deviation of 1.38. This indicates mixed results of participants learning something new, which may be attributed to a participant’s previous experience, with approximately half of participants having prior knowledge of or having trained for the Mabu stance previously. Very similar results were observed for Survey Item 2.7 (M = 3.67, SD = 1.15) and Survey Item 2.8 (M = 3.75, SD = 1.29), where there were mixed results among participants in having an educational experience due to the surveyed previous knowledge of the Mabu stance. We hypothesized that the results for the set of instrumental attitude survey items may have been higher if the participants examined had no prior martial arts experience.

Survey Items 2.9 and 2.10 are related to gaming continuance intention. The results for Survey Item 2.9 had a very positive mean score of 4.25 with a standard deviation of 0.75, highlighting the aspect that most participants had an enjoyable experience overall. Additionally, the results of Survey Item 2.10 showed a mean score of 4.08 with a standard deviation of 0.67, which demonstrates that most participants would like to play more of the developed Kung Future Game long term. Participants of the experiments mentioned that they would continuously use the system on average 2–3 times a week. Furthermore, most participants also responded that they would use the system for long-term remote training if it included additional techniques for practicing, such as the various stances in Kung Fu or their respective martial arts. This is positive confirmation of our design goal in creating an educational, engaging, and enjoyable user experience for long-term remote training.

The final survey was the user feedback on gameplay and interaction, which included only open-ended questions. Survey Item 3.1 asked each participant about his/her interactions with the virtual coach in the game. There were 11 out of the 14 participants who responded positively towards interacting with the virtual avatar and found it useful in learning Mabu and keeping them motivated. One participant expressed a preference for traditional methods of martial arts training and felt like a virtual avatar could not supplement in-class training. The remaining two participants had a neutral interaction, with both mentioning that more-detailed feedback from the coach would have made their interactions more positive. This is interesting feedback, as in the design of the app, we targeted a beginner-friendly interaction with simple feedback so as not to overwhelm the user during training. However, based on the user feedback, our developed system may be refined in the future by adding a level of in-game feedback or guidance based on the proficiency or previous training experience of the user. This can be fine tuned in the in-game settings to allow for a more-personalized game experience. This also relates to the responses to Survey Items 3.2 and 3.3, where all participants expressed that the virtual coach’s postural feedback was useful and they would like to see more modes of feedback in-game. The most-requested form of additional feedback was vocal feedback from the coach to give detailed information on how to correct their form. This is an interesting concept in game design and needs to be carefully designed and implemented. It may be useful to receive vocal feedback if it is informative, moderate, and not repetitive. For the case of Mabu, it may be considered a form of meditation, despite performing an isometric exercise. In this case, a traditional practitioner may not favor the use of real-time vocal feedback and find it disturbing to his/her training. However, when first learning martial arts stances or techniques, vocal feedback may definitely be beneficial and can be a clear indicator when on-screen postural feedback can be hard to visualize, such as when in the middle of a form.

Survey Items 3.4 and 3.5 polled participants on what they liked the most and what they liked the least about their experience. The responses from the participants for 3.4 included the instant balance detection, game interface, gamification aspects such as the leaderboard and competing with others, and the virtual coach’s posture feedback. It was positive to observe that all participants enjoyed the gamification elements and were impressed with the capabilities of the LifeMat system in detecting their real-world balance and relaying that to in-game events. This adds to the validation of our system in regard to the research focus of this paper in creating an IoT platform for self-supported martial arts training. The results of Survey Item 3.5 included responses on the need for more-detailed feedback, the single mode of Mabu training, and the need to calibrate the device. In terms of system calibration, it was an important step for the experiments to ensure an accurate Mabu stance could be verified and tracked for each user depending on his/her body shape. Although the one-time calibration took approximately 10 s per user at the start of the experiment, it may be viewed as cumbersome for long-term continued use. In this case, a more-simplistic algorithm for detecting the Mabu stance without calibration can be implemented and will provide instantaneous entry into the training game upon startup. Many of the responses from Survey Item 3.5 were followed up by answers to Survey Item 3.6. Some participants would improve the game by adding additional modes of training and deeper gamification elements such as training with mini games.

Survey Item 3.7 questioned the participants about whether or not they would recommend the Kung Future game to their friends. All 14 participants responded with “Yes”, which is a positive reflection of the overall user experience. Additionally, Survey Item 3.8 had positive feedback from all 14 participants and included responses mentioning the usefulness of the game in learning basic martial arts techniques, but with some critiquing the need to supplement the system with real-world classes. Our proposed IoT training system is certainly recommended as a supplementary tool to in-class training or training with a professional human martial artist. However, our proposed IoT training system aims to benefit beginners as an introductory training tool to build up confidence to join a martial arts class or to be used as a smart training tool for all martial artists to examine their technique when undertaking solo training to pick up on elements that may not be evident to the human eye. Furthermore, it is also worth noting other answers to Survey Item 3.8, where participants from other martial arts expressed a newfound interest in learning Kung Fu after taking part in the Kung Future experiment. This validates our research goal of developing a system to help users gain access and learn new skills in a comfortable self-paced setting. Finally, Survey Item 3.9 asked participants on how they would compare traditional training with gamified remote training using our system. The responses showed mixed results with many participants once again appreciating the system as an introductory and long-term supplementary training device, but no substitute for traditional classes. Two participants explained that they enjoyed traditional classes due to socializing with others and exchanging techniques. Four participants mentioned the need for occasional feedback from a human trainer to directly review and correct them. However, six participants mentioned the advantage of an IoT sensor system in being able to detect fine changes in balance that may not be apparent to humans. Furthermore, the use of an IoT-game-based system allows for fully independent, remote training at any time without the need for a coach, which may result in accelerated training results.

One of our main research objectives was to solve a problem with remote training where the practitioner is unsure if he/she is performing a technique correctly or not without the presence of a coach. Especially in martial arts, there are no current solutions to our knowledge that allow a practitioner to receive instant and accurate feedback on his/her technique. This is the basis for the development of the Kung Future app. Based on the feedback performance study and survey results, we were able to evaluate our research question and demonstrate that our IoT sensing platform, LifeMat and our Serious Game Kung Future, was highly effective for remote martial arts training. During the gamification study, the increased motivation and engagement of all participants was clearly observed when performing Mabu with the game feedback active. There was also the added motivation of keeping the virtual Master on the platform as long as possible by maintaining the correct technique in the real world.

Prior to this study, we hypothesized that gamification may only be effective in some areas as the effect and appeal may become repetitive over time. Therefore, we had to take into consideration the motivation of the user for both short-term learning value and long-term gain.

According to an article by *Black Belt Magazine*, one of the world’s leading magazines, on the use of gamification in martial arts [44], examples of daily life gamification include grades in our schools, rank in the military, and promotion/salary in business. This reward-type improvement system is called operant conditioning and is a method of learning through the use of rewards and punishments. They go on to mention that, if a reward system (gamification) is adopted, then it is important that the rewards continue in order to keep practitioners motivated, unless the ultimate goal of the training provides real-world value that surpasses the reward-based system. A prime example of this is in martial arts, where the ability to protect one’s self holds more value than the satisfaction of endlessly advancing rewards [44]. Since participants in our study may have felt a stronger sense of real-world gain when using the Kung Future system, this may have accounted for the positive score for long-term use. Additionally, the use of gamification elements such as the leaderboard and level-up system contributed to the high motivation of the participants and high score for long-term continued use.

## 5. Conclusions

Our results demonstrated that our system could effectively educate and train a user in the Shaolin Kung Fu Mabu stance. The feasibility experiments highlighted the capabilities of the LifeMat system in sensing balanced and imbalanced postures with high sensitivity and accuracy. From the feedback experiments, we observed that the game feedback mode (GFB) had the highest performance results among participants when compared to training without any feedback and when training with clock-based feedback. User feedback from the surveys indicated that the system rated highly in terms of engagement, motivation, long-term continued usage, and intuitiveness. The use of gamification was highly effective in assisting new users to learn a basic, yet fundamental martial arts stance, and our system demonstrated promise to be extended to further applications. Although some participants mentioned the need for other forms of feedback and more detailed insight, these can be implemented in future versions as customization options. These initial results highlight the potential for such a system to enhance human skill learning through gamification and active feedback through an IoT sensing platform and Serious Game. Furthermore, our system can be used to introduce people to a new sport or martial art in a more-comfortable and -flexible manner, especially for those who may be hesitant to join a public class.

Based on the feedback performance study and survey results, we were able to evaluate our research question and demonstrate that our IoT sensing platform, LifeMat and our Serious Game Kung Future, was highly effective for remote martial arts training and can be used for training of a technique when a professional is not present. In future research, we will aim to further develop our platform for Serious Games in order to breath new life into ancient martial arts and other sports and fitness activities by leveraging the latest advances and research in IoT and AI technologies.

## Figures and Tables

**Table 1:** Serious Game experience survey.

QID	Survey Question	M (SD)
2.1	I can keep winning at computer games for a long time.	3.33 (1.23)
2.2	I can figure out most computer games.	4.50 (0.67)
2.3	No matter how hard I try, I do not do well when playing computer games.	2.00 (0.85)
2.4	I had an entertaining time.	4.58 (0.51)
2.5	The experience was enjoyable.	4.33 (0.65)
2.6	I learned something new.	3.50 (1.38)
2.7	I had an educational experience.	3.67 (1.15)
2.8	The experience was informative.	3.75 (1.29)
2.9	I enjoyed training with the game feedback more than without feedback.	4.25 (0.75)
2.10	I would like to play more Kung Future in the future.	4.08 (0.67)

**Table 2:** Participant background survey.

QID	Survey Question	Results
1.1	What is your Age Group?	MODE = 24–29-year-old group
1.2	What is your Gender?	Male = 11, female = 3
1.3	What is your Height (cm)?	M = 172.83, SD = 8.19
1.4	What is your Weight (kg)?	M = 83, SD = 23.79
1.5	Do you play video games?	Yes = 14, No = 0
1.6	What is your fitness activity level?	Low = 0, moderate = 11, high = 3
1.7	Do you practice or have you ever practiced any Martial Art?	Yes = 14, No = 0
1.8	Do you play any sport? If so which sport?	Yes = 6, No = 8

**Table 3:** User feedback on gameplay and interaction survey.

QID	Survey Question	Dimension
3.1	What do you think of the virtual coach in the game?	Adaptivity
3.2	What do you think of the feedback used for training?	Adaptivity
3.3	Would you like to see other modes of feedback?	Adaptivity
3.4	What did you like the most?	Gaming Experience
3.5	What did you like the least?	Gaming Experience
3.6	How would you change the game so that you enjoy it more?	Gaming Experience
3.7	Would you recommend a game like this to your friends?	Gaming Continuance
3.8	What do you think about learning martial arts through this game?	Learning Experience
3.9	How would you compare learning martial arts through this game with learning in a traditional class?	Learning Experience

## Data Availability

The data presented in this study is available within the article.

## Supplementary Materials

The following supporting information can be downloaded at: https://www.mdpi.com/article/10.3390/s23177565/s1, Video S1: Training Gamification User Study.

## Abbreviations

The following abbreviations are used in this manuscript:

IoT	Internet of Things
SBC	Single-Board Computer
PCB	Printed Circuit Board
GFB	Game Feedback
CFB	Clock Feedback
NFB	No Feedback

## References

[1] Draeger D., Khim P., Bennett A. (2020). Shaolin Kung Fu: The Original Training Techniques of the Shaolin Lohan Masters.

[2] Chen H., Zuo Y., Law R., Zhang M. (2021). Improving the Tourist’s Perception of the Tourist Destinations Image: An Analysis of Chinese Kung Fu Film and Television. Sustainability.

[3] Zhouxiang L. (2019). A History of Shaolin: Buddhism, Kung Fu and Identity.

[4] Shahar M. (2017). The Shaolin Monastery: History, Religion, and the Chinese Martial Arts.

[5] Zhu W.G., Lin Q.C.B. (2004). Shaolin wu shu qi yuan wu Zhong “chuang chuan shuo” ping shu [A commentary on the five originations of Shaolin martial arts]. Beijing Ti Yu Da Xue Xue Bao.

[6] Wang G. (2012). Chinese Kung Fu.

[7] Meyer M.J., Molle A., Judkins B.N., Bowman P. (2021). Martial arts in the pandemic. Martial Arts Stud..

[8] Su C.H., Cheng C.H. (2015). A mobile gamification learning system for improving the learning motivation and achievements. J. Comput. Assist. Learn..

[9] Kuo-Deemer M. (2019). Qigong and the Tai Chi Axis: Nourishing Practices for Body, Mind, and Spirit.

[10] Kit G.W.K. The Horse Stance. https://shaolin.org/general-2/horse-stance.html.

[11] Matthews M., Matthews H., Yusuf M., Doyle C. (2016). Traditional martial arts training enhances balance and neuromuscular control in female modern martial artists. J. Yoga Phys. Ther..

[12] Yang J.-M. (1996). The Essence of Shaolin White Crane: Martial Power and Qigong.

[13] Kee Y.H. (2019). Looking East for mindfulness: A glimpse of practices and research on shaolin martial arts and related practices to advance sport psychology. Psych.

[14] Falk B., Mor G. (1996). The effects of resistance and martial arts training in 6-to 8-year-old boys. Pediatr. Exerc. Sci..

[15] Tao S., Chuang T., Chen W. Investigating effects of game-based design mechanisms on learners’ reasoning ability: A cluster analysis. Proceedings of the 26th International Conference on Computers in Education, ICCE 2018.

[16] Yang J.C., Chen C.H., Jeng M.C. (2010). Integrating video-capture virtual reality technology into a physically interactive learning environment for English learning. Comput. Educ..

[17] Sevinç D., Çolak M. (2019). The effect of electronic body protector and gamification on the performance of taekwondo athletes. Int. J. Perform. Anal. Sport.

[18] Ishac K., Eager D. (2021). Evaluating martial arts punching kinematics using a vision and inertial sensing system. Sensors.

[19] Nor N.N., Sunar M.S., Kapi A.Y. User experience of gamified virtual reality (VR) in sport: A review. Proceedings of the Science and Technologies for Smart Cities: 5th EAI International Summit, SmartCity360.

[20] Entertainment S.I. (2006). EyeToy: Kinetic Combat. [GAME]. http://www.eyetoykinetic.com/.

[21] Ubisoft (2013). Fighter Within. [GAME]. http://fighter-within.ubi.com/fighter-within/en-GB/home/.

[22] Paliyawan P., Sookhanaphibarn K., Choensawat W., Thawonmas R. Body motion design and analysis for fighting game interface. Proceedings of the 2015 IEEE Conference on Computational Intelligence and Games (CIG).

[23] Clark R.A., Bryant A.L., Pua Y., McCrory P., Bennell K., Hunt M. (2010). Validity and reliability of the Nintendo Wii Balance Board for assessment of standing balance. Gait Posture.

[24] Holmes J.D., Jenkins M.E., Johnson A.M., Hunt M.A., Clark R.A. (2013). Validity of the Nintendo Wii® balance board for the assessment of standing balance in Parkinson’s disease. Clin. Rehabil..

[25] Park D.S., Lee G. (2014). Validity and reliability of balance assessment software using the Nintendo Wii balance board: Usability and validation. J. Neuroeng. Rehabil..

[26] Baranyi R., Willinger R., Lederer N., Grechenig T., Schramm W. Chances for Serious Games in rehabilitation of stroke patients on the example of utilizing the Wii Fit Balance Board. Proceedings of the 2013 IEEE 2nd International Conference on Serious Games and Applications for Health (SeGAH).

[27] Prosperini L., Fortuna D., Giannì C., Leonardi L., Marchetti M.R., Pozzilli C. (2013). Home-based balance training using the Wii balance board: A randomized, crossover pilot study in multiple sclerosis. Neurorehabilit. Neural Repair.

[28] Sparrer I., Duong Dinh T.A., Ilgner J., Westhofen M. (2013). Vestibular rehabilitation using the Nintendo® Wii Balance Board–a user-friendly alternative for central nervous compensation. Acta-Oto-Laryngol..

[29] Hämäläinen P., Ilmonen T., Höysniemi J., Lindholm M., Nykänen A. Martial arts in artificial reality. Proceedings of the SIGCHI Conference on Human Factors in Computing Systems.

[30] Hsu W.C., Shih J.L. (2017). Applying augmented reality to a mobile-assisted learning system for martial arts using kinect motion capture. Blended Learning: Concepts, Methodologies, Tools, and Applications.

[31] Jan Y.F., Tseng K.W., Kao P.Y., Hung Y.P. Augmented Tai-Chi Chuan Practice Tool with Pose Evaluation. Proceedings of the 2021 IEEE 4th International Conference on Multimedia Information Processing and Retrieval (MIPR).

[32] Kao P.Y., Han P.H., Jan Y.F., Yang Z., Li C.H., Hung Y.P. On Learning Weight Distribution of Tai Chi Chuan Using Pressure Sensing Insoles and MR-HMD. Proceedings of the VR.

[33] Bourahmoune K., Ishac K., Amagasa T. (2022). Intelligent Posture Training: Machine-Learning-Powered Human Sitting Posture Recognition Based on a Pressure-Sensing IoT Cushion. Sensors.

[34] Ishac K., Suzuki K. A smart cushion system with vibrotactile feedback for active posture correction. Proceedings of the Haptic Interaction: Science, Engineering and Design 2.

[35] Ishac K., Suzuki K. (2018). Lifechair: A conductive fabric sensor-based smart cushion for actively shaping sitting posture. Sensors.

[36] Bourahmoune K., Amagasa T. AI-powered Posture Training: Application of Machine Learning in Sitting Posture Recognition Using the LifeChair Smart Cushion. Proceedings of the IJCAI.

[37] Bourahmoune K., Ishac K., Carmichael M., Amagasa T. Owro: A Novel Robot For Sitting Posture Training Based On Adaptive Human Robot Interaction. Proceedings of the 2022 IEEE International Conference on Big Data (Big Data).

[38] Eager D., Ishac K., Zhou S., Hossain I. (2022). Investigating the Knuckleball Effect in Soccer Using a Smart Ball and Training Machine. Sensors.

[39] LifeMat (2023). Lifeform AI. https://lifeformai.com/.

[40] Unity (2023). Unity Technologies. https://unity.com.

[41] Law E.L.C., Sun X. (2012). Evaluating user experience of adaptive digital educational games with Activity Theory. Int. J. Hum.-Comput. Stud..

[42] Moizer J., Lean J., Dell’Aquila E., Walsh P., Keary A.A., O’Byrne D., Di Ferdinando A., Miglino O., Friedrich R., Asperges R. (2019). An approach to evaluating the user experience of Serious Games. Comput. Educ..

[43] Ketelhut D.J. (2011). Assessing gaming, computer and scientific inquiry self-efficacy in a virtual environment. Serious Educational Game Assessment.

[44] Pruim A. (2023). Gamification of the Martial Arts. https://blackbeltmag.com/gamification-martial-arts.

